# Non-ionising UV light increases the optical density of hygroscopic self assembled DNA crystal films

**DOI:** 10.1038/s41598-017-06884-8

**Published:** 2017-07-26

**Authors:** Alexandria E. Gasperini, Susy Sanchez, Amber L. Doiron, Mark Lyles, Guy K. German

**Affiliations:** 1 Department of Biomedical Engineering, Binghamton University, Binghamton, NY 13902 USA; 2 Office of the Vice President for Research, University of Rhode Island, Kingston, RI 02881 USA

## Abstract

We report on ultraviolet (UV) light induced increases in the UV optical density of thin and optically transparent crystalline DNA films formed through self assembly. The films are comprised of closely packed, multi-faceted and sub micron sized crystals. UV-Vis spectrophotometry reveals that DNA films with surface densities up to 0.031 mg/mm^2^ can reduce the transmittance of incident UVC and UVB light by up to 90%, and UVA transmittance by up to 20%. Subsequent and independent film irradiation with either UVA or UVB dosages upwards of 80 J/cm^2^ both reduce UV transmittance, with reductions scaling monotonically with UV dosage. To date the induction of a hyperchromic effect has been demonstrated using heat, pH, high salt mediums, and high energy ionising radiation. Both hyperchromicity and increased light scattering could account for the increased film optical density after UV irradiation. Additional characterisation of the films reveal they are highly absorbent and hygroscopic. When coated on human skin, they are capable of slowing water evaporation and keeping the tissue hydrated for extended periods of time.

## Introduction

Deoxyribonucleic Acid (DNA) is a long chain biopolymer that has been studied extensively in order to understand its relevance to human health. Studies examining the effect of ultraviolet (UV) light irradiation on DNA photodamage^1^ have shown that damage to both mitochondrial and genomic DNA can lead to painful sunburn^2^, photoageing^3, 4^ and a degradation in skin barrier function^5^. UV light has also been shown to be the most prominent carcinogen in our natural environment^1, 6^. UV irradiation can cause two types of DNA lesion: cyclobutane pyrimidine dimers^7^ and 6–4 photoproducts^1^. Both of these lesions distort the molecular structure: introducing bends and kinks and thereby impeding transcription and replication^8^.

The UV spectrum is typically divided into 4 regions^9, 10^: UVA (315–400 nm, 3.94–3.10 eV), UVB (280–315 nm, 3.94–4.43 eV), UVC (200–280 nm, 6.20–4.43 eV) and vacuum-UV (100–200 nm, 6.20–12.40 eV). UVA, UVB and UVC light all fall outside of the range considered ionising; the lower limit being 10 eV^11^. UVC and vacuum-UV are fully attenuated by Earth’s atmospheric gases^6, 12, 13^ and studies have shown only limited evidence of UVC carcinogenicity in humans^14^. However, both UVB and UVA have been associated with increased risk of skin cancer^15^. Incidences of cutaneous malignant melanoma, squamous cell carcinoma and basal cell carcinoma increase with ambient exposure to UV light^6^. Due to their combined presence in solar light however, the relative contribution of UVA and UVB light to DNA photodamage continues to be a topic of debate^1, 7, 10, 16–20^.

In addition to healthcare related research, considerable advances have been made in exploiting DNA self-assembly to create a diverse range of three dimensional architectures for applications in physics, biology and chemistry^21–26^. Thin DNA films have been shown to exhibit novel electrical^27–29^, optical^22, 30–36^ and biosensing properties^22, 37–39^. Associated work on liquid DNA solutions has also revealed that hyperchromicity, an increase in the optical density of a material, can be induced by denaturing DNA molecules with heat, pH, ionising radiation and high salt mediums^40–42^. To date however, the impact of non-ionising UV light on the photonic properties of DNA molecules and self-assembled structures they can form remains unclear. In this article, we take a first step towards understanding how non-ionising UV irradiation alters the photonic properties of thin, self-assembled DNA films. Spectrophotometry is used to quantify changes in the transmission, absorbance, and transmittance of films after independent irradiation with UVA and UVB light. Additional swelling and evaporation kinetics of films coating human *stratum corneum* skin tissue are quantified to further characterise their structure and hygroscopic properties.

## Results

### Characterising DNA film structure

**Figure 1:**
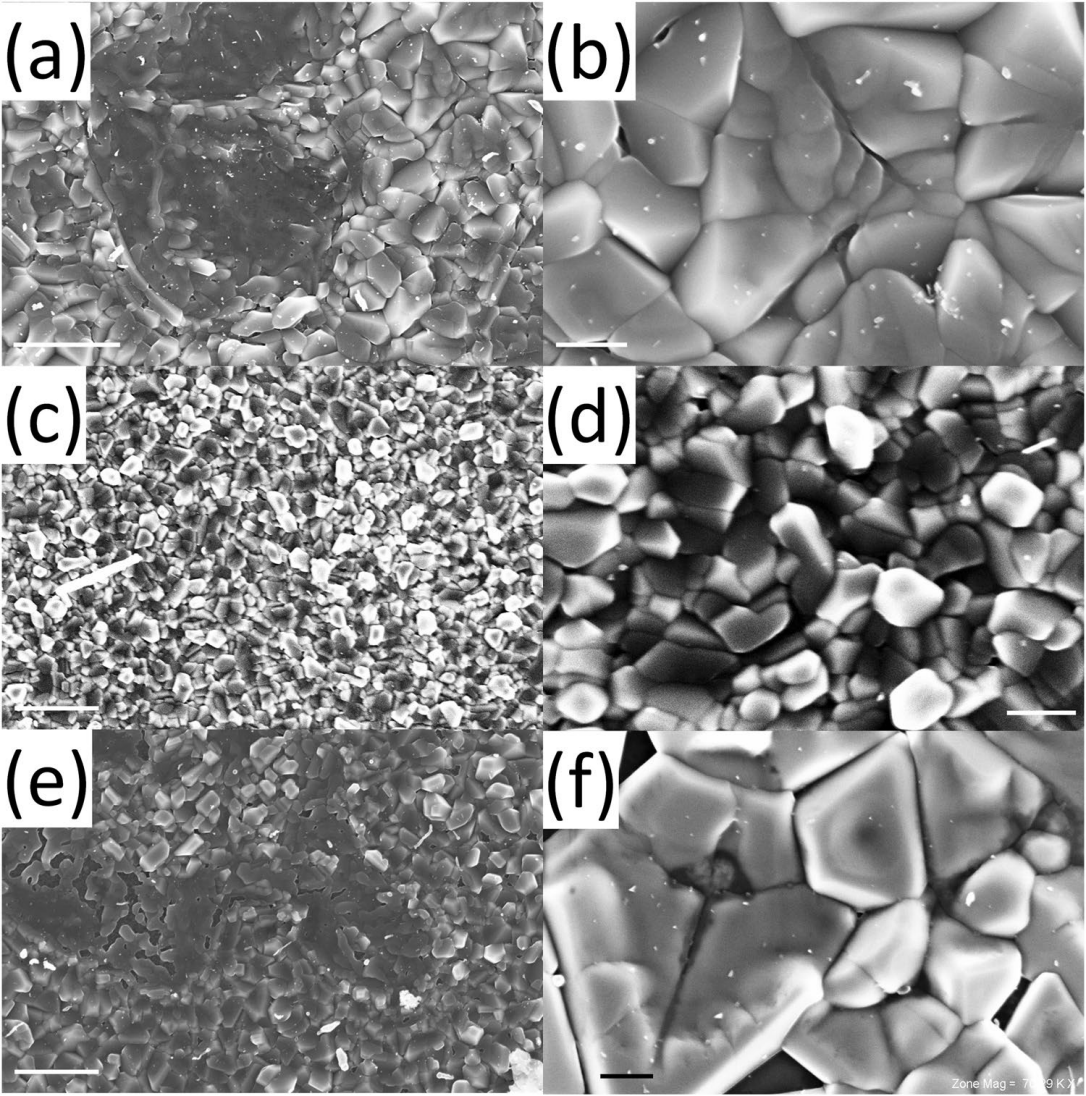
DNA film structure. Scanning electron micrographs of (**a,b**) a 0.0031 mg/mm^2^ surface density DNA film exposed only to ambient UV light, (**c,d**) a 0.0031 mg/mm^2^ DNA film irradiated with a dosage of 320 J/cm^2^ UVA light (365 nm), and (**e,f**) a 0.0031 mg/mm^2^ DNA film irradiated with a dosage of 320 J/cm^2^ UVB light (302 nm). Scale bars in the left and right columns of images respectively denote 2 μm and 400 nm.

We first characterise the structure of films formed by drying aqueous DNA solutions on a glass coverslip. At macroscopic length scales, all the films appear homogeneous, however scanning electron microscopy (SEM) reveals that the films exhibit topographical heterogeneities at sub-micron length scales. Figure 1(a) and (b) show scanning electron micrographs of a 0.0031 mg/mm^2^ surface density DNA film that has only been exposed to ambient light. The film is comprised of closely packed, discrete and multi-faceted crystals. Figure 1(c) and (d) show SEM images of a 0.0031 mg/mm^2^ film after exposing it to a dosage of 320 J/cm^2^ UVA light (peak narrowband emission at 365 nm). Figure 1(e) and (f) show complementary images of a 0.0031 mg/mm^2^ film after exposure to an equivalent dosage of UVB light (peak narrowband emission at 302 nm). Table 1 details the average crystal size for UVA and UVB irradiated 0.0031 mg/mm^2^ surface density films along with 0.0031 mg/mm^2^ and 0.0124 mg/mm^2^ surface density control films exposed only to ambient light. Both UVA and UVB exposure induce statistically significant reductions in crystal size relative to films exposed only to ambient light, however the greatest reduction occurs with UVA irradiation. Increasing the film density also increases the average crystal size.

**Table 1:** DNA Film Crystal size variation with surface density and UV irradiation.

Film surface density (mg/mm^2^)	UV dosage	Average crystal size (nm)	Statistical Significance
0.0031	Ambient light	578 ± 184 (n = 105)	
0.0031	UVA 320 J/cm^2^	354 ± 122 (n = 128)	0.0031 mg/mm^2^ (Ambient vs. UVA) [p < 0.0001]
0.0031	UVB 320 J/cm^2^	522 ± 161 (n = 101)	0.0031 mg/mm^2^ (Ambient vs. UVB) [p = 0.0302]
0.0124	Ambient light	811 ± 281 (n = 128)	Ambient (0.0031 vs. 0.0124 mg/mm^2^) [p < 0.0001]

Crystal size measurements for each surface density and UV dosage are extracted from a minimum of n = 2 films. Statistical differences in crystal size with UV exposure or film surface density were quantified using a nonparametric, two-tailed Mann-Whitney test.

### Characterising the photonic properties of DNA films

**Figure 2:**
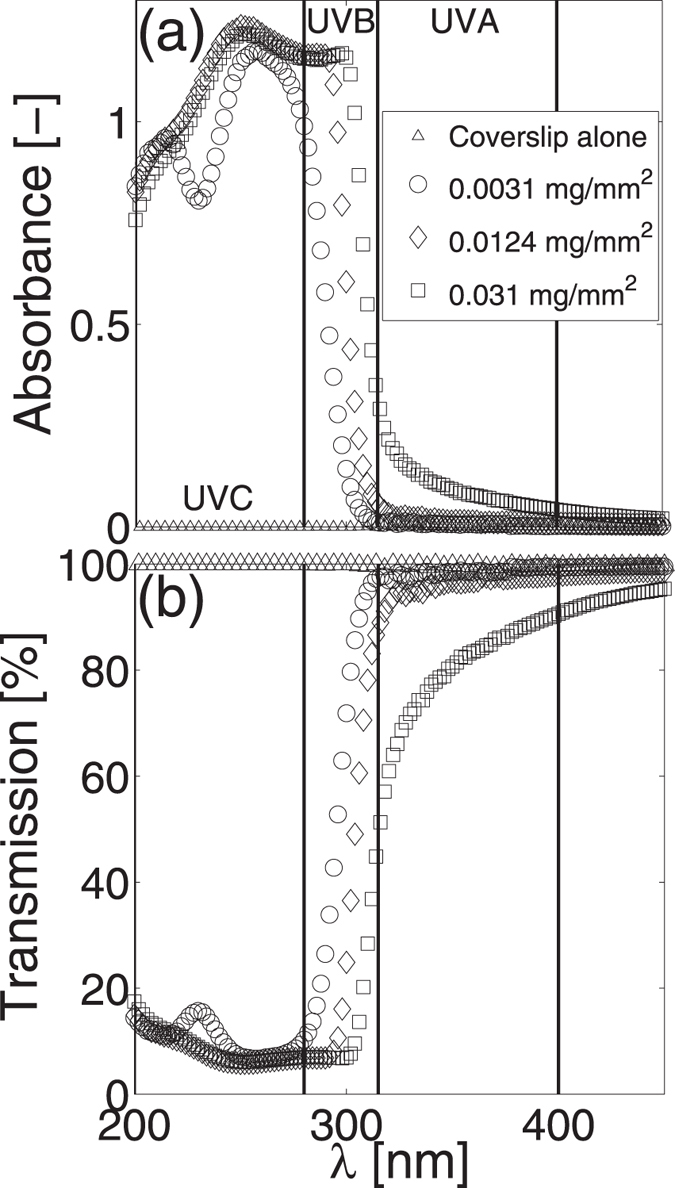
Surface density effects on UV absorbance and transmission. Average (**a**) absorbance and (**b**) transmission spectra of DNA films with surface densities of 0.0031 mg/mm^2^ (n = 3, open circle), 0.0124 mg/mm^2^ (n = 3, open diamond) and 0.031 mg/mm^2^ (n = 3, open square) plotted against wavelength in the range ${\rm{200}}\le \lambda \le {\rm{450}}$ nm. Open triangle symbols denote the average (n = 3) absorbance and transmission spectra of the optically transparent glass coverslips on which all DNA films are coated. Vertical lines delineate the UVA (315–400 nm), UVB (280–315 nm), and UVC (200–280 nm) ranges. Standard deviations in all cases are smaller than the symbol size and have not been plotted to improve visual clarity.

We next interrogate the photonic properties of DNA films coated on UV transparent coverslips by measuring their absorbance and transmission spectra. Figure 2(a) shows that the films do not absorb light at wavelengths greater than 500 nm, therefore measured spectra are truncated to show only UV and visible blue wavelengths. All of the films absorb UV light strongly in the UVC range (200–280 nm) and exhibit an abrupt reduction in absorbance over the UVB range (280–315 nm). Increases in film surface density delay the onset of this transition to longer UVB wavelengths. Within the UVA range (315–400 nm), 0.0031 and 0.0124 mg/mm^2^ surface density films absorb little light, however 0.031 mg/mm^2^ films show notable UVA absorbance. Figure 2(b) highlights that all films reduce UVC transmission by greater than 90% at certain wavelengths. Increases in film surface density result in decreased transmission in the UVA, UVB, and UVC ranges. The most notable changes occur within the range ${\rm{300}}\le \lambda \le {\rm{450}}$ nm.

### Characterising changes in DNA film photonic properties with UV irradiation

**Figure 3:**
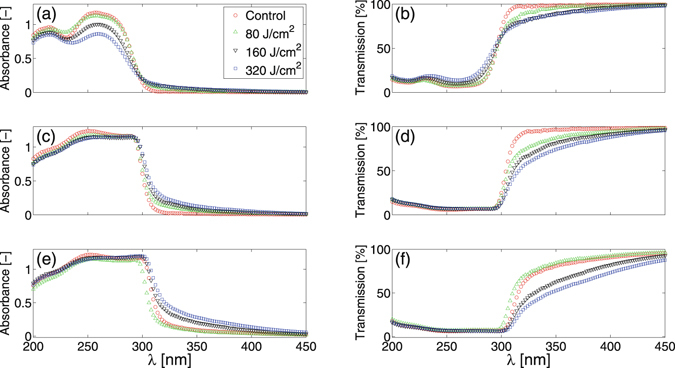
Effects of UVB irradiation. Absorbance (left column) and transmission (right column) plotted against wavelength, λ, for films with a surface density of 0.0031 mg/mm^2^ (top row), 0.0124 mg/mm^2^ (middle row) and 0.031 mg/mm^2^ (bottom row). Each of the panels (**a**–**f**) display the average (n = 3 individual films for each dosage) absorbance or transmission spectrum prior to UVB irradiation (Control: red circle) and after UVB dosages of 80 J/cm^2^ (green triangle), 160 J/cm^2^ (black inverted triangle) and 320 J/cm^2^ (blue square). Standard deviations in all cases are smaller than the symbol size and have not been plotted to improve visual clarity.

Changes in the photonic properties of DNA films after UV irradiation are subsequently assessed. The absorbance and transmission spectra of films only exposed to ambient light are first captured. The films are then sequentially irradiated with increasing dosages of either UVA or UVB light using a UV lamp. After each dosage, the film spectra are re-quantified. The UV lamp increases the local air temperature around the films to no greater than 27 °C, substantially below the temperature required to denature DNA^43^ (>80 °C). Figure 3 shows changes in the average absorbance and transmission spectra of DNA films after exposure to UVB dosages of 80, 160 and 320 J/cm^2^ (n = 3 individual films for each dosage). A UVB dosage of 160 J/cm^2^ has previously been reported to damage the barrier function of human *stratum corneum* ^5^ skin tissue. As shown in Fig. 3(a) and (b), increases in UVB dosage cause 0.0031 mg/mm^2^ films to increase transmission and decrease absorbance within the UVC range. A small decrease in UVA transmission is also observed. In contrast, films with surface densities of 0.0124 and 0.031 mg/mm^2^, shown in Fig. 3(c) through (f), show little variation in UV transmission at wavelengths less than 300 nm, but notable decreases at longer wavelengths. Reductions in UV transmission scale monotonically with UVB dosage except 0.031 mg/mm^2^ films irradiated with a 80 J/cm^2^ dosage. Relative to controls, this dosage causes a small decrease in transmission around 300 nm. Decreases in UVA transmission with UVB dosage become more pronounced as film surface densities increase.

**Figure 4:**
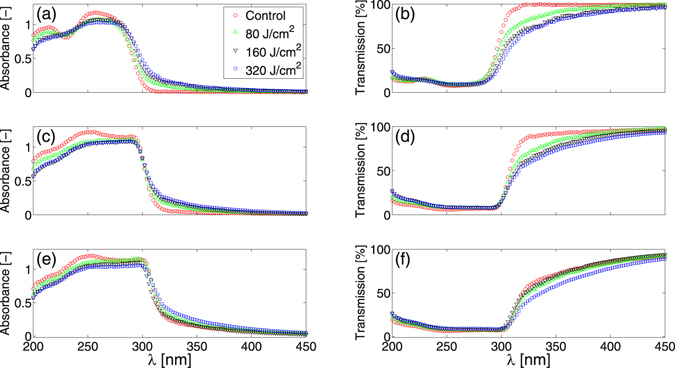
Effects of UVA irradiation. Absorbance (left column) and transmission (right column) plotted against wavelength, λ, for films with a surface density of 0.0031 mg/mm^2^ (top row), 0.0124 mg/mm^2^ (middle row) and 0.031 mg/mm^2^ (bottom row). Each of the panels (**a**–**f**) display the average (n = 3 individual films for each dosage) absorbance or transmission spectrum prior to UVA irradiation (Control: red circle) and after UVA dosages of 80 J/cm^2^ (green triangle), 160 J/cm^2^ (black inverted triangle) and 320 J/cm^2^ (blue square). Standard deviations in all cases are smaller than the symbol size and have not been plotted to improve visual clarity.

Figure 4 shows variations in the absorbance and transmission spectra of films with different surface densities after irradiation with UVA dosages of 80, 160 and 320 J/cm^2^. Increases in UVA dosage for the 0.0031 mg/mm^2^ films result in notable decreases in UV transmission at wavelengths greater than 300 nm and small decreases in absorbance at wavelengths below 300 nm. In contrast with the UVB irradiation results in Fig. 3, reductions in UVA and UVB transmission with UVA dosage become less pronounced with increases in film surface density.

**Figure 5:**
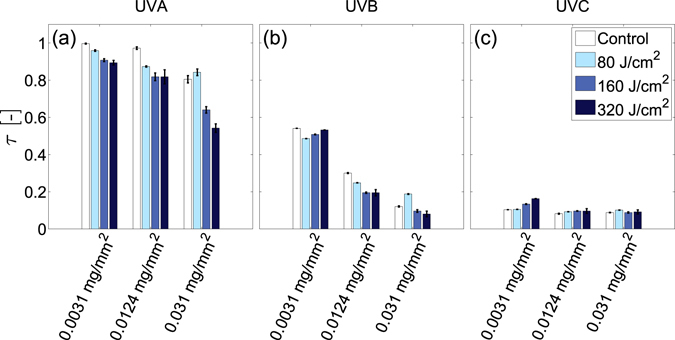
Effect of UVB dosage on UV film transmittance. Average DNA film transmittance, τ, in the (**a**) UVA range (315–400 nm), (**b**) UVB range (280–315 nm) and (**c**) UVC range (200–280 nm) prior to UVB exposure (open bar) and after UVB irradiation with dosages of 80, 160 and 320 J/cm^2^ (respectively denoted by increasingly dark bar colors). Error bars denote standard deviations of n = 3 individual films.

In order to characterise overall changes in UV transmission through the films after UVA or UVB irradiation, the fraction of total incident light transmitted, or transmittance^44^, $\tau ={\int }_{{\lambda }_{{\rm{\min }}}}^{{\lambda }_{{\rm{\max }}}}\tau (\lambda ){{\rm{\Phi }}}_{\lambda i}d\lambda /{\int }_{{\lambda }_{{\rm{\min }}}}^{{\lambda }_{{\rm{\max }}}}{{\rm{\Phi }}}_{\lambda i}d\lambda$, is quantified over the UVA (${\lambda }_{\max }=400$ nm, ${\lambda }_{\min }=315$ nm), UVB (${\lambda }_{\max }=315$ nm, ${\lambda }_{\min }=280$ nm) and UVC (${\lambda }_{\max }=280$ nm, ${\lambda }_{\min }=200$ nm) ranges. Here, ${{\rm{\Phi }}}_{\lambda i}$ denotes the incident spectral flux and $\tau (\lambda )$ denotes the spectral transmittance; the ratio of the transmitted spectral flux to the incident spectral flux at each wavelength. Figure 5(a–c) respectively show how the transmittance of light in the UVA, UVB and UVC ranges differ with film surface density and UVB dosage. Increases in UVB dosage cause notable reductions in transmittance primarily within the UVA range. Relative to controls exposed only to ambient light, the 0.031 mg/mm^2^ films exhibit the largest decrease from τ = 0.76 ± 0.05 to τ = 0.64 ± 0.02 and τ = 0.54 ± 0.02 after UVB dosages of 160 and 320 J/cm^2^ respectively. The uncertainties here correspond to standard deviations. Smaller changes in UVA transmittance are observed for films with lower surface densities. Dosages of 320 J/cm^2^ cause transmittance reductions of Δτ = −0.1 in 0.0031 mg/mm^2^ films and Δτ = −0.13 in 0.0124 mg/mm^2^ films. UVB dosages of 160 J/cm^2^ or greater also marginally reduce UVB transmission for 0.0124 and 0.031 mg/mm^2^ films. Small increases in UVC transmittance are observed only for the 0.0031 mg/mm^2^ films.

**Figure 6:**
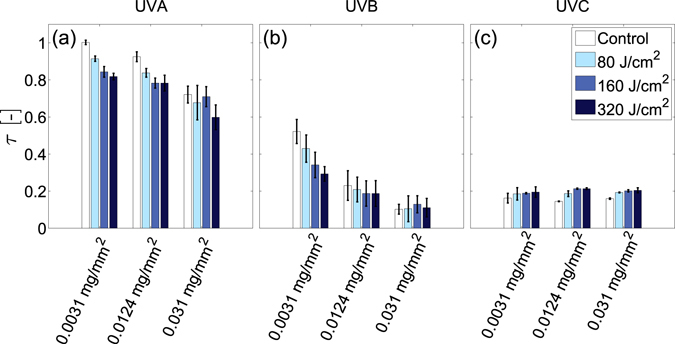
Effect of UVA dosage on UV film transmittance. Average DNA film transmittance, τ, in the (**a**) UVA range (315–400 nm), (**b**) UVB range (280–315 nm) and (**c**) UVC range (200–280 nm) prior to UVA exposure (open bar) and after UVA irradiation with dosages of 80, 160 and 320 J/cm^2^ (respectively denoted by increasingly dark bar colors). Error bars denote standard deviations of n = 3 individual films.

Figure 6(a), (b) and (c) respectively show how the transmittance of light in the UVA, UVB and UVC ranges differ with UVA dosage. With a dosage of 320 J/cm^2^, fractional UVA film transmittance decreases by Δτ = −0.18, −0.16, and −0.16 for the 0.0031, 0.0124, and 0.031 mg/mm^2^ surface density films respectively. Notable decreases in UVB film transmittance also occur, but only for the 0.0031 mg/mm^2^ film, where a dosage of 320 J/cm^2^ reduces UVB transmittance by Δτ = −0.24. UVA irradiation also marginally increases UVC transmission for films with surface densities of 0.0124 mg/mm^2^ or greater. Dosages of 80 J/cm^2^ increase UVC transmittance by $0.06\le {\rm{\Delta }}\tau \le 0.1$, however subsequent irradiation does not alter transmittance further.

### Characterising DNA film swelling and evaporation kinetics

**Figure 7:**
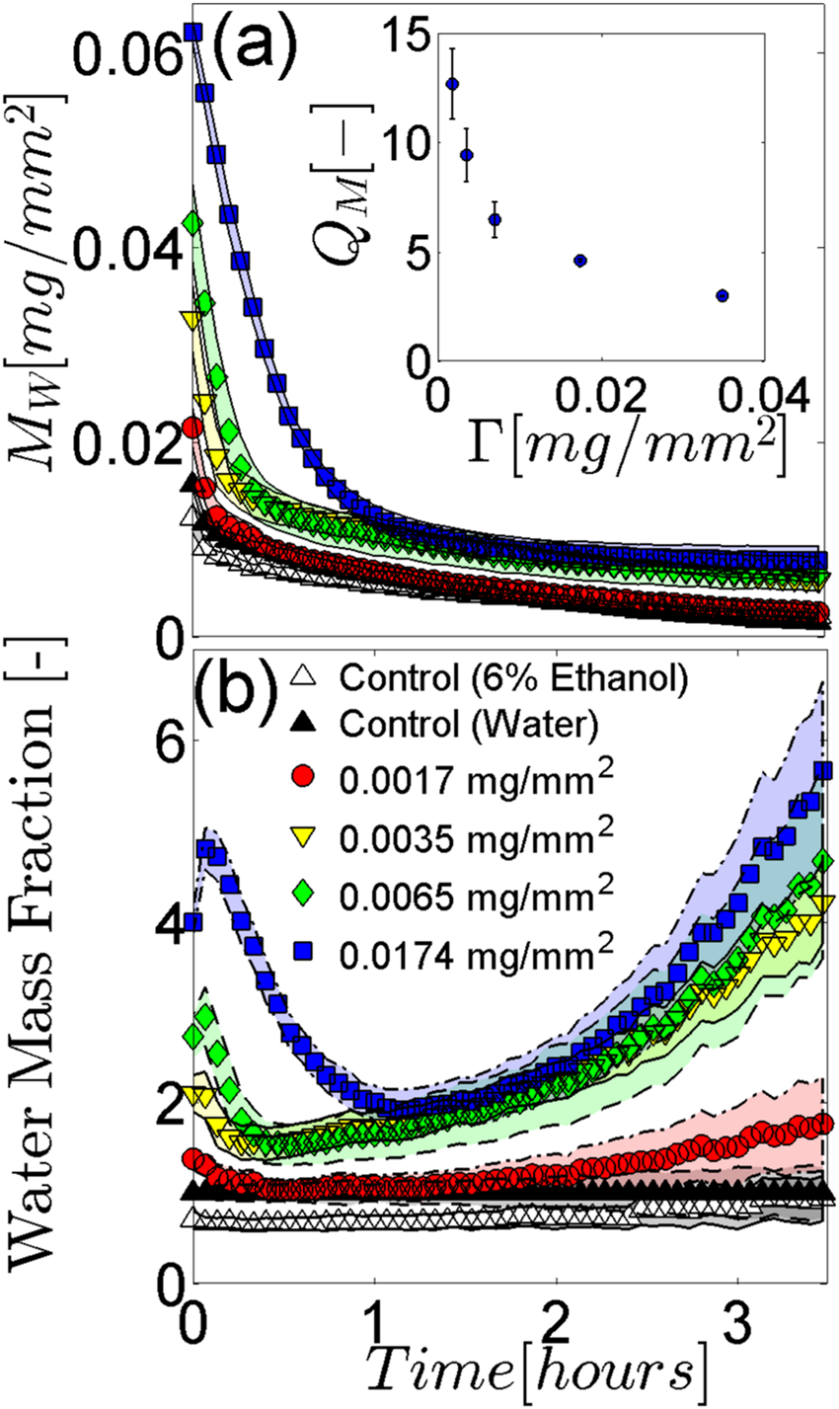
Evaporation kinetics of DNA film coated SC. (**a**) Average area scaled water mass, *M*_*W*_, of control and DNA film coated SC samples drying over time in a 24% R.H. environment. Control results include SC samples coated with DIW water (black triangle) and 6% aqueous ethanol solutions (white triangle). DNA film coating results include surface densities of 0.00173 (red circle), 0.00347 (yellow inverted triangle), 0.00694 (green diamond), and 0.0174 mg/mm^2^ (blue square). Shaded regions denote standard deviations of n = 3 individual SC samples. (Inset) Average mass swelling ratio, *Q*_*m*_, plotted against DNA film surface density, $\Gamma$. Error bars denote standard deviations of n = 3 individual SC samples. When not visible, errors are smaller than the symbol size. (**b**) Results from panel (**a**) scaled by the DIW coated control results (black diamond). Shaded regions denote propagated standard deviations.

Studies exploring the evaporation kinetics of DNA films when topically applied to human skin are additionally performed to further probe their structure and hygroscopic properties. Isolated samples of identically sized human *stratum corneum* (SC, surface area A = 1440 mm^2^), the outermost layer of skin that governs trans epidermal water loss^45^ are adhered to an elastomer coated aluminium substrate. Samples are then uniformly coated with 500 μl of an aqueous DNA solution, then desiccated for 24 hr at 10% R.H to establish the total mass of SC sample, DNA film and substrate, *M*_0_. Once fully rehydrated, changes in the sample mass, *M*(*t*), are recorded over a 3.5 hr period within a controlled 24% relative humidity (R.H.) environment. This period is sufficiently long to observe drying behavior^46, 47^. Figure 7(a) displays changes in the area scaled average water mass of the SC and DNA films over time, ${M}_{W}(t)=(M(t)-{M}_{0})/A$. The fully hydrated area scaled water mass, *M*_*W*_(0), increases with film surface density. Subsequent drying over the first 60 min causes all coated and uncoated samples to rapidly dehydrate. Thereafter, the rate of drying decreases. After the first 20 min of drying, samples with 0.00173 mg/mm^2^ coatings show no difference in *M*_*W*_ relative to control samples coated only with deionized water (DIW). Higher surface density films however result in a larger *M*_*W*_ throughout the 3.5 hr drying period. In order to quantify the effects of the ethanol used to create the DNA solutions, drying behavior of SC samples coated with 6% aqueous ethanol solutions are also assessed. These samples exhibit a reduced water mass relative to DIW coated controls over the first 2 hr of drying. Thereafter, they become comparable. This indicates that the ethanol reduces rather than increases the ability of SC to hold water in a hydrated state.

The inset figure in Fig. 7(a) plots changes in the DNA film mass swelling ratio, *Q*_*M*_, against film surface density, Γ. This ratio denotes the quotient of the maximum swollen DNA film mass to the dry mass of the DNA film alone^48^. The swollen mass of the DNA films are obtained by subtracting the average fully hydrated mass of uncoated SC samples from the average hydrated mass of SC samples coated with the DNA films. This swelling ratio is commonly used to characterise hydrogel behavior, where swelling of the hydrogel is eventually balanced by the elastic restoring forces of the crosslinked polymer network^48^. The data indicates that as Γ increases, *Q*_*M*_ decreases and the maximum area scaled water mass that the film can absorb is approached. To verify this trend, the swelling ratio of 0.0348 mg/mm^2^ films are additionally measured and included in the results.

To further highlight the impact of the DNA films on the rate of cutaneous water loss, Fig. 7(b) shows the results of Fig. 7(a) scaled by the average area scaled water mass of DIW coated controls. Over the first 60 min of drying, samples coated with a DNA film exhibit increased rates of evaporation relative to controls. This rapid evaporation quickly eliminates the elevated water masses within the fully hydrated film coated SC samples. The rate of this evaporation also increases with film surface density. Thereafter, the evaporation rate of samples coated with a 0.0017 mg/mm^2^ film remains near consistent with control samples. However, the three highest surface density films exhibit water evaporation rates smaller than SC samples without a film coating. This diminished rate of evaporation also scales monotonically with the film surface density. These results are consistent with previous studies of drying behavior in polymeric films^49^. For intermediate and high concentrations of a polymer in a solvent, the diffusion coefficient of the solvent across the drying film, which is directly related to the evaporation rate, decreases as the polymer concentration is increased. In contrast, as the polymer concentration is decreased towards very dilute systems, the diffusion coefficient plateaus and becomes consistent with that of the solvent alone. Relative to controls, the time varying change in water evaporation rate of the three highest surface density films after 60 min drying also suggests that as water evaporates, the DNA mass fraction within the films increase, further slowing drying.

## Discussion

Previous research exploring the material and optical properties of DNA based materials is extensive, with numerous reviews on the subject^24–26^. However, a majority of this research focuses on DNA-surfactant complexes and structurally modified DNA. Studies exploring the photonic properties of unmodified DNA crystal films formed through evaporation of aqueous DNA solutions^31, 33, 35, 36, 50^ have studied their absorbance, transmission, refractive index anisotropy, and circular dichroism. This research has revealed the impact of UV-light, magnetic fields and fluid surface tension on the resultant DNA film structure. To date however, studies examining changes in UV transmission and absorbance of films after irradiation with UVA and UVB light have not been reported.

Hyperchromicity, or an increase in the optical density of a material, can be induced in liquid DNA solutions by denaturing DNA molecules with temperature, high-salt mediums, ionising radiation and pH^40–42^. In this article, we demonstrate that similar increases in the optical density of thin, self assembled DNA films can be induced through irradiation with sufficient dosages of non-ionising UVB (Fig. 5) and UVA (Fig. 6) light. Figures 3 and 4 collectively show that increases in both film surface density and UV light exposure notably increase the optical density of the films in the UVB and UVA regimes. Increases in DNA crystal size with film surface density, as shown in Fig. 1 and Table 1, could account for greater UV attenuation due to increased transmission path lengths. However, an increased path length would not explain the UV induced changes in optical density because prolonged UV irradiation decreases the average size of crystals (Table 1). The inset graph in Fig. 7 (a) provides some insight into the DNA film structure. As the film surface density is increased, the maximum mass swelling ratio is approached. This characteristic is observed in polymeric hydrogels, and is attributed to a rise in crosslinking density as the polymer concentration is increased^48, 51, 52^. The DNA used to create the films is partially denatured and likely to form interchain bonds similar in structure to crosslinked hydrogels. The inclusion of ethanol in the preparation of the films may also induce physical crosslinks between DNA molecules. Ethanol is known to change DNA conformation and enable increasingly complex molecular morphologies to form, such as loops^53^. These could promote the physical entanglement of DNA chains. To our knowledge, films formed in this manner have not been previously examined. A rise in crosslinking density that would increase interactions between light and the DNA network could alternatively explain why increased UV attenuation occurs at higher film surface densities. This mechanism would also explain the UV induced increases in optical density. Both UVA and UVB light have previously been demonstrated to induce DNA lesions^1, 7, 16, 17, 54^. These lesions would enable the molecules to directly absorb more UV light^40–42^. Additionally, the distortions could also induce conformal changes to the crystalline film structure; leading to an increased crosslinking density and a higher UV attenuation via increased light scattering. Indeed, previous studies have revealed that UVC light exposure increases the molecular weight of DNA films^35^; suggesting that the number of intermolecular crosslinks within the film structure increases.

Currently, it remains unclear whether the underlying cause of UV induced optical density variations within the films occurs via an increase in the direct absorbance of UV light by distorted DNA molecules, or an increase in light scattering from a more entangled polymer network. Both of these mechanisms could account fully, or in combination, to the observed increases in optical density. To evaluate relative contributions from each mechanism, future work should relate variations in crystal structure and density with UV dosage, perhaps measured by glancing angle synchrotron X-ray diffraction. Circular dichroism spectroscopy could also elucidate changes in macromolecular conformation with UV dosage.

The combined ability of the DNA films to: attenuate UV light (Fig. 2) while remaining optically transparent, increase UV attenuation with prolonged UV exposure (Figs 3–6), keep human skin tissue hydrated for extended periods of time (Fig. 7(a)) and reduce the water evaporation rates from the coated skin tissue (Fig. 7(b)) suggests their potential use as an alternative biocompatible wound covering. A DNA coating could enable continual wound monitoring without the need to remove opaque dressings that would expose the injured tissue to the external environment. It would also provide protection from UV photodamage^10^ and a moist environment known to promote faster wound healing rates^55–57^.

## Methods

### Preparation of DNA solutions

Aqueous DNA solutions were prepared by coating the sides of a glass vial with DNA (Deoxyribonucleic acid sodium salt from salmon sperm, not highly polymerised, MP Biomedicals LLC, molecular weight ~6 × 10^6^), then wetting the DNA with pure ethanol (Sigma-Aldrich, St Louis, MO). The wetted DNA was then agitated in deionised water (DIW, Milli-Q Integral 15, EMD Millipore, Billerica, MA) until homogeneous. Samples with concentrations of 0.5, 1, 2.0, 5.0, and 10.0% DNA by mass in DIW were prepared.

### Preparation of glass slides for spectrophotometry

A 22 × 22 × 1 mm Viosil fused silica glass coverslip (Quartz Scientific, Fairport Harbor, OH) was rinsed with pure ethanol to remove residual contaminants. Once fully dried, 300 μL of DIW or an aqueous DNA solution was pipetted on to the slide, ensuring even coverage. DNA films created using 0.5, 2 and 5% aqueous DNA solutions respectively produced films with surface densities of 0.0031, 0.0124, and 0.0310 mg/mm^2^. Slides were allowed to dry in a sealed petri dish containing a desiccant (size 6 Mesh Drierite, Fisher Scientific, Waltham, MA) for 24 hr, resulting in a solid crystalline DNA film.

### Ultraviolet treatment

The DNA film side of coated glass coverslips were illuminated from above using a UV Lamp (8 Watt EL, UVP, Upland, CA) with stand. Samples were exposed to UVB (302 nm narrowband) or UVA (365 nm narrowband) light for periods equating to light dosages of 80, 160, and 320 J/cm^2^. For samples positioned 76 mm from the light source, the average intensities of the UVA and UVB lamp bulbs are 1.5 mW/cm^2^ and 1.6 mW/cm^2^ respectively. Exposure times for the two UV ranges were varied in order to irradiate the DNA films to the same dosage. For example, a dosage of 80 J/cm^2^ required an exposure time of 14.81 hours for UVA, while 13.88 hours was required for UVB. Films exposed only to ambient light were used as controls. A hygrometer with probe (445815, Extech Instruments, Nashua, NH) was used to record the temperature in the vicinity of the films throughout the exposure period.

### Measurement of DNA film absorbance and transmission spectra

Coated glass coverslips were loaded in a UV-Vis-NIR Spectrophotometer (Lambda 950, Perkin Elmer, Waltham, MA) with the DNA film coating facing the aperture. Absorbance and transmission spectra (n = 3 individual samples for each DNA film density) were recorded over the wavelength range 175–750 nm.

### Measurement of DNA film crystal size

The maximum breadths of discrete DNA crystals were extracted from scanning electron micrographs (Zeiss SUPRA 55, Oberkochen, Germany) of the films and used to establish a mean and standard deviation. The normality of the measured breadths for each film was established by performing a D’Agostino & Pearson normality test. Discrete crystal sizes were not normally distributed. As such, statistical differences in crystal size with UV exposure or film surface density were quantified using a nonparametric, two-tailed Mann-Whitney test. A p-value of less than p ≤ 0.05 was considered statistically significant. All statistical analyses were performed using GraphPad Prism 7.00.

### Preparation of elastomer substrates for drying studies

Aluminium foil was wrapped around 60 × 24 mm glass coverslips (Fisherbrand, Fisher Scientific, Pittsburgh, PA). A silicone elastomer base (Sylgard 184; Dow Corning, Midland, MI) mixed with its curing agent in the ratio 55:1 was mixed, degassed, and then spin coated on each aluminum substrate at 2000 r.p.m for 65 sec. Substrates were then cured for 24 hr in laboratory conditions (23 °C, 40% relative humidity (R.H.)).

### Preparation and deposition of Stratum Corneum

Full thickness skin (42 and 49 yrs. female breast) was received from elective surgery. An exempt approval (3002–13) was obtained to perform research using de-identified tissue samples pursuant to the Department of Health and Human Services (DHHS) regulations, 45 CFR 46.101(b)(4). The choice of breast tissue was based on attainability from surgery. Isolation of the superficial *stratum corneum* (SC) tissue was achieved using a standard water bath and trypsin solution based technique^58^. After separation, the SC was allowed to dry to laboratory conditions on plastic mesh for 24 hr. An elastomer coated aluminium substrate was removed from the glass slide and cut to a dimension of 24 × 60 mm using a scalpel and metal rule. The mass of the elastomer coated aluminum substrate was then recorded using an analytical balance (MS104S, Mettler Toledo, Ithaca, NY) with 0.1 mg resolution. The underside of an isolated SC sheet was then laminated to the elastomer substrate, ensuring that no bubbles became trapped. Once laminated, the SC was trimmed to the size of the substrate. The prepared slide was then placed in a humidity chamber containing a desiccant (size 6 Mesh Drierite) for 24 hr. Relative humidity conditions of 10% were recorded in the sealed container using a hygrometer. After drying, the mass of each desiccated SC and substrate was recorded.

### Coating SC samples with DNA films

For control treatments, SC samples were coated with 500 μL of either DIW or DIW containing 6% pure ethanol by volume. This corresponded to the maximum concentration of ethanol used to enable dissolution of the DNA. For DNA film deposition, SC samples were coated with 500 μL of a DNA solution. The coating process was performed by pipetting small drops on to the SC in a grid pattern. This ensured complete and even coverage of the SC surface. Samples were then desiccated in a humidity chamber with a recorded humidity of 10% R.H. for 24 hr. Upon drying, solutions with DNA concentrations of 0.5, 1, 2, 5 and 10% respectively formed thin solid films with average surface densities of 0.00173, 0.00347, 0.00694, 0.0174 and 0.0348 mg/mm^2^.

### Measurement of Evaporation kinetics

The dry mass of each SC sample, DNA film and substrate, *M*_0_, was recorded using an analytical balance. Samples were subsequently transferred to an environmental chamber with recorded humidity of 100% R.H for 48 hr to rehydrate. These drying and wetting stages ensured that no unbound water accumulated on the SC surface, improving repeatability. Hydrated samples were individually placed within a glass enclosure on top of an analytical balance. A humidity control system^59^ equilibrated the air within the glass enclosure to 24 ± 5% R.H. Mass measurements were recorded every 30 seconds for 3.5 hr. To eliminate mass drift effects, the balance was tared every 10 min by lifting the sample off the weighing pan, taring the balance, and replacing the sample. This process did not alter the humidity conditions within the glass enclosure. Aluminium foil was used as a substrate instead of a glass coverslip to minimise mass drift from electrostatic charges.

### Data Availability

The data that support the findings of this study are available from the corresponding author upon reasonable request.
